# Access to communication support for community-dwelling people with dementia: A mixed methods study exploring local perspectives within the United Kingdom context

**DOI:** 10.1093/ageing/afaf150

**Published:** 2025-06-03

**Authors:** Anna Hockley, Deborah Moll, Jemima Littlejohns, Zoe Collett, Catherine Henshall

**Affiliations:** University College London - Department of Language and Cognition, Chandler House 2 Wakefield Street, London WC1N1PF, England, United Kingdom of Great Britain and Northern Ireland; National Institute for Health and Care Research (NIHR), Oxford Health Clinical Research Facility, Oxford Health NHS Foundation Trust, Warneford Hospital, Warneford Lane, Oxford, OX3 7JX, England, United Kingdom of Great Britain and Northern Ireland; Oxford Health NHS Foundation Trust, Research and Development, Warneford Hospital, Warneford Lane, Oxford, OX3 7JX, England, United Kingdom of Great Britain and Northern Ireland; Oxford Health NHS Foundation Trust, Memory and Cognition Research Delivery Team, Warneford Hospital, Warneford Lane, Oxford, OX3 7JX, England, United Kingdom of Great Britain and Northern Ireland; Oxford Health NHS Foundation Trust, Research and Development, Warneford Hospital, Warneford Lane, Oxford, OX3 7JX, England, United Kingdom of Great Britain and Northern Ireland; Oxford Institute of Applied Health Research (OxInAHR), Oxford Brookes University, Headington Campus, Oxford, OX3 0BP, England, United Kingdom of Great Britain and Northern Ireland; Nursing and Midwifery, National Institute for Health and Care Research, 60 Cannon Street, London, EC4N 6NP, England, United Kingdom of Great Britain and Northern Ireland

**Keywords:** Communication, Dementia, Quality-of-life, Service provision, Speech and language therapy, Qualitative Research, Older people

## Abstract

**Introduction:**

Communication difficulties occur in all dementia subtypes. This can affect individuals’ identity, relationships, and quality-of-life of people with dementia and their relatives. Professional guidelines recommend access to communication intervention, for example through speech and language therapy (SLT) services, but anecdotal evidence suggests that the type and availability of this provision varies.

**Aims:**

This study aimed to explore the communication needs of people with dementia, their quality-of-life impact, and local and national service provision.

**Methods:**

This mixed-methods study comprised three phases:

1. An online survey of UK SLTs (n = 74) examined the type and availability of communication intervention for people with dementia.
2. Semi-structured interviews with people with dementia (n = 7) and relatives (n = 9) explored experiences of communication difficulties and support received.
3. Two focus groups with local NHS Trust staff including: SLTs (n = 7), and dementia HCPs (n = 6).

Data was analysed using descriptive statistics and thematic analysis.

**Results:**

Four main themes were identified: dementia-related communication changes; accessing support for communication; identifying communication strategies; and service considerations. The survey identified inconsistent or absent communication input for many people with dementia. Interview and focus group findings highlighted diverse dementia-related communication needs which impacted quality-of-life and support required. Participants suggested service-level considerations for addressing these needs.

**Conclusion:**

Dementia-related communication impairments are diverse and can considerably impact quality-of-life of people with dementia and their relatives. Communication intervention service-provision varies widely, resulting in unmet needs. These findings add to the growing evidence-base on dementia-related communication difficulties, and highlight a need to develop clinical services.

## Key Points

- Dementia-related communication impairments impact quality-of-life for people with dementia and their relatives.
- Communication input for this population varies across services, often resulting in unmet needs.
- Service barriers include: inconsistent commissioning, prioritising dysphagia caseloads, limited awareness of speech and language therapy (SLT).
- Joined-up, individualised post-diagnostic care when planning communication support for this population is imperative.
- The findings highlight the need to develop and implement equitable and sustainable service-delivery models.

## Introduction

Dementia results from damage to nerve cells in the brain. This can be caused by different diseases, which result in varying dementia subtypes [1]. Dementia symptoms are progressive, and include difficulties in areas such as memory, thinking, learning, orientation and communication [2]. There is currently no cure for dementia. Dementia’s prevalence is increasing, affecting approximately 55 million people globally, including 520,000 in the United Kingdom (UK) [3–6]. There is a growing focus on promoting quality-of-life and ‘living well’ with dementia [7–10].

### Communication and dementia

Communication impairments are associated with all dementia subtypes [11]. However, the nature, severity, and progression vary significantly, both across dementia subtypes and between individuals [2]. Impairments can include: difficulty expressing and understanding language (aphasia); motor speech impairments (dysarthria); cognitive communication difficulties (e.g. difficulty concentrating, retaining information or staying on topic); and reading and writing difficulties [2, 12]. These difficulties can impact social interactions, relationships, occupations, employment and leisure activities, and can lead to increased behavioural and psychological symptoms of dementia [13–17]. Communication often becomes more challenging as the disease progresses, and people can lose the ability to communicate their thoughts and needs [12, 17, 18]. Dementia-related communication difficulties are considered among the most frequent and challenging experiences for families impacted by dementia, with considerable quality-of-life implications [19–22].

### Speech and language therapy within dementia care

The National Collaborating Centre for Mental Health [23] committed to improving post-diagnostic dementia support. They suggested access to non-pharmacological interventions and communication skills training for relatives and caregivers of people with dementia (PWD) [20]. Furthermore, The Royal College of Psychiatrists’ Memory Services National Accreditation Programme [24] states that communication input should be provided when needed and suggests that memory services have access to speech and language therapy (SLT).

Speech and language therapists (SLTs) specialise in treating communication impairments. Royal College of Speech and Language Therapists’ [21] guidance states that SLT in dementia care should be collaborative and multidisciplinary, and may include: supporting diagnosis; direct communication-focused interventions; identifying compensatory communication strategies; and communication partner training. It advises equitable input for communication impairment. Despite this, communication difficulties are considered one of the largest unmet needs within dementia care [21]. The Unmet Needs Model [25, 26] suggests that dementia-related communication impairments lead to increased unmet needs, and further research has found a high prevalence of unmet needs relating to social contact and meaningful activities [27]. Commissioning for communication input is variable and sometimes absent, resulting in a UK-wide ‘postcode lottery’ and gaps in service provision [15, 21]. The reasons for and quality-of-life impact of this variable and inequitable service provision requires further exploration, to inform priorities for commissioners and service providers.

### Aims

This study explored the perspectives of UK-wide SLTs on the nature of dementia communication service provision. It then explored the perspectives of PWD, their relatives, and National Health Service (NHS) staff in one UK locality, to gain a deeper understanding of the impact of limited services.

## Methods

### Study design

This study comprised the following phases:

Phase 1: A national online survey of SLTs.

Phase 2: Semi-structured interviews with PWD and their relatives.

Phase 3: Focus groups with SLTs and dementia HCPs.

It used an iterative sequential mixed methods approach. Researchers formulated an initial framework of questions to be proposed at each phase, using deductive reasoning. Data was analysed after each phase, informing refinement of the framework for the next phase, in conjunction with patient and public involvement (PPI) input. PPI contributors were people with lived experience of dementia (had a diagnosis of dementia or were a relative of someone living with dementia) accessed through local third-sector organisations or the local NHS Trust’s communications channels. Online meetings about the research study took place during study design, between study phases one and two, and at the end of data collection. Contributors’ input supported the design of user-friendly patient information sheets, guidance for user-friendly remote interviewing, amendment of staff focus group questions, highlighting priority areas to write up for publication, and accessible results dissemination. PWD were supported to participate in PPI activities through options to engage via online meetings or via email, accessible presentations during meetings, and support from their third-sector organisation staff member. Study phase one explored national service delivery; this informed phases two and three, which focused on the local context. Ethical approval (reference 21/SC/0135) was gained.

### Rigour

This study involved triangulation by including a range of perspectives and methodologies, increasing its rigour [28, 29]. The researchers came from various professional backgrounds, and PPI contributors with a range of experiences were consulted throughout. These varied perspectives supported reflexivity, increasing rigour through framing the study findings through a collection of insights and perspectives.

### Phase 1: Survey of SLTs

#### Recruitment and data collection

To capture data on the types of service delivery models offered nationally, a cross-sectional, online, anonymous survey for SLTs was developed, based on Volkmer et al. [30]. The survey included closed and open-ended (free text) questions, and focused on service provision for PWD, unmet needs and barriers to intervention access (Appendix 1). Work setting and professional experience information was collected. The survey was piloted by SLTs in one NHS Trust and revised to improve usability and acceptability. The final survey was hosted on Microsoft Forms and distributed via social media and the RCSLT’s mailing list. Responses from 50–100 SLTs were sought. SLTs were eligible if they were UK-based registered clinicians (Table 1). An initial screening question asked if the respondent had ‘ever seen a patient with confirmed or queried dementia’; if not, the survey ended at that point. The survey was open for 4 weeks (November to December 2021).

**Table 1:** **Eligibility criteria.** Population approached for each phase of the study

		Inclusion criteria	Exclusion criteria
**Phase 1:** SLT^1^ survey participants		• SLT	
		• Currently working with adults in the United Kingdom^2^	
**Phase 2:** patient and caregiver interviews			
	*For patient*	• Diagnosis of any type of dementia	• Lacking capacity to consent
		• Participant feels they are experiencing changes to speech, language, or communication as a result of their dementia	• Speech, language, or communication difficulties not primarily caused by their dementia
		• Participant is willing and able to give informed consent for participation in the study	• Communication difficulties so severe that they are unable to participate in audio recorded interviews
		• Must be able to understand English well enough to interview	
		• Aged 18 years or above	• Under 18 years of age
		• Living at home	• Living in residential care
	*For caregiver*	• Must consider themselves an informal caregiver of a person with dementia	• Unable to participate in audio recorded interviews
		• Must be in regular contact with the person with dementia who feels they are experiencing changes to communication as a result of their dementia (*minimum of weekly*)	• Are not currently in regular contact with a person with dementia, who is experiencing difficulties with communication
		• Participant is willing and able to give informed consent for participation in the study	• Informal caregiver of person with dementia who is living in residential care
		• Must be able to understand English well enough to interview	• Carers who are formally hired and paid
		• Aged 18 years or above	
		• The patient is living at home	
**Phase 3:** staff focus groups		• Staff working within the local NHS Trust who work with patients in the community experiencing dementia in any capacity OR SLTs^1^ employed by local NHS Trust working with adults (*SLTs don’t have to necessarily be working with PWD*)	• Unable/unwilling to participate for 1 hour in a focus group
		• Participant is willing and able to give informed consent for participation in the study	
		• Aged 18 years or above	

Note: ^1^Speech and language therapist ^2^National Health Service

#### Data analysis

Data from closed text responses were analysed using descriptive statistics. Free text, open ended response data was analysed thematically [31].

### Phase 2: Semi-structured interviews with PWD and relatives

*Recruitment and Data Collection.*

PWD and their relatives were identified through purposive sampling. They were invited to participate in semi-structured interviews, either individually or as dyads. PWD were eligible if they had a dementia diagnosis (any subtype), were living at home, and were experiencing dementia-related communication changes (Table 1). Relatives were eligible if they were in weekly contact with a person who met the above criteria. Participants were excluded if they were unable to give informed consent to participate, if primary communication changes were not dementia-related, or if these difficulties precluded participation in audio-recorded interviews.

Participants were recruited from one NHS Trust in South England, which does not currently commission communication interventions for PWD. Participants were identified through clinician referrals, third-sector organisations, and searches of clinical records. Two researchers conducted screening and made initial contact (JL, ZC). Eligible individuals were invited for a videocall or telephone interview (according to participant preference) with a researcher (AH, DM, JL, ZC).

Semi-structured interviews were conducted via telephone or videocall (March–June 2022) and were audio-recorded using an encrypted Dictaphone. Informed consent was audio-recorded prior to interview commencement. Demographic information was collected (Table 3). Interviews followed a semi-structured topic guide, which was informed by PPI feedback and Phase 1 findings. Topics included communication changes, communication support/intervention, and quality-of-life implications (Appendix 2).

The researchers aimed to recruit 16 participants (with roughly equal numbers of PWD and relatives), after which time data saturation was achieved.

### Phase 3: Focus groups with SLTs and HCPs

#### Recruitment and data collection

Five SLTs, and five other HCPs who routinely saw community-dwelling PWD, were purposively sampled to participate in two separate online focus groups (March 2023) (Table 1). Groups were divided into SLTs and other HCPs to facilitate context-specific discussions and develop ideas for service-level improvements. Participants were recruited via the local NHS Trust’s communication channels.

Participants provided written informed consent and demographic information. Focus groups were facilitated by two researchers (AH, JL) via videocall. A semi-structured topic guide (Appendix 3) was followed, informed by PPI feedback, findings from phases 1–2, and a recent systematic review [32]. Questions focused on the need for communication input, and service delivery models. Focus groups were audio-recorded using an encrypted Dictaphone.

### Data analysis of phases 2 and 3

Interviews and focus groups were transcribed by an approved transcription company, who removed all patient identifying information. Researchers reviewed the transcripts for completeness and the removal of any remaining identifying information, and to familiarise themselves with the data. Transcripts were analysed by two researchers using a combination of inductive and deductive thematic analysis [31] (phase 2: ZC, DM; phase 3: ZC, JL). Four researchers (AH, DM, JL, ZC) discussed emerging themes and considered the extent to which they aligned with and enriched the survey findings, before producing a final narrative summary.

## Results

The themes identified across the survey, interviews and focus groups pertained to both service considerations and the impact of communication difficulties on daily life and well-being. This paper focuses on service considerations.

## Participants

### Phase 1: Survey

Seventy-five SLTs consented to the online survey, with 74 respondents eligible following screening. Respondents were located across the UK and were mainly employed by the NHS in Band 6 or 7 roles (Table 2). Most respondents had more than 10 years’ SLT experience, mainly worked in general adult services (n = 44, 60%), with 19 (26%) working in specialist dementia or mental health settings, 6 (8%) in neurology and 5 (7%) in learning disabilities.

**Table 2:** Demographic of participant population of phase one, a national online survey of SLTs

		Responses (n = 74)
Years post qualification	Newly qualified	4 (5.4%)
	1-2 yrs	5 (6.8%)
	3-4 yrs	4 (5.4%)
	4-10 yrs	20 (27%)
	+10 yrs	41 (55.4%)
NHS banding	5	6 (8.1%)
	6	29 (39.2%)
	7	27 (36.5%)
	8	11 (14.9%)
	Other	1 (1.4%)
Geographic location	Southwest England	4 (5.4%)
	Southeast England	13 (17.6%)
	London	13 (17.6%)
	East England	3 (4.1%)
	West Midlands	5 (6.8%)
	East Midlands	5 (6.8%)
	Yorkshire and The Humber	10 (13.5%)
	Northwest England	7 (9.5%)
	Northeast England	4 (5.4%)
	Ireland	2 (2.7%)
	Wales	5 (6.8%)
	Scotland	3 (4.1%)
Healthcare service *(note some responders worked across multiple settings)*	Acute health care	22 (29.7%)
	Mental health care	17 (23%)
	Primary care	22 (29.7%)
	Charity/third sector	1 (1.4%)
	Community health care	2 (2.7%)
	Outpatient rehabilitation	11 (14.9%)
	Inpatient rehabilitation	2 (2.7%)
	Unspecified	1 (1.4%)
Have you seen patients with dementia for…?	Swallowing	7 (9.5%)
	Communication	1 (1.4%)
	Both	66 (89.2%)
Are you working in a service that specialises in dementia and mental health, or a general adult service?	Specialist in dementia and mental health	19 (25.7%)
	General adult	44 (59.5%)
	Neurology (including stroke)	6 (8.1%)
	Learning disabilities	5 (6.8%)

Key survey results are available in Appendix 4.

### Phase 2: Interviews

Seven PWD and nine relatives were interviewed. This consisted of six dyads interviewed together, one dyad interviewed separately, and two individual relatives (Table 3).

**Table 3:** Demographic of participant population for phase two, patient and relative interviews

Patient group (n = 7)	Age:	55–64	1
		65–74	-
		75+	6
	Gender:	Male	4
		Female	3
	Ethnicity:	White – English	4
		White – British	2
		White – European	1
	Dementia diagnosis:	Alzheimer’s disease	3
		*Logopenic variant = 1 participant*	
		Mixed Alzheimer’s & Vascular	1
		Fronto-temporal dementia	3
		*Semantic variant = 1 participant*	
		Lewy body disease	1
		Other sub-type	1
		*Primary progressive aphasia = 1 participant*	
Caregiver group (n = 9)	Age:	55–64	2
		65–74	5
		75+	2
	Gender:	Male	2
		Female	7
	Ethnicity:	White – English	4
		White – British	4
		White – Other	1
	Relationship:	Spouse	8
		Adult children	1

### Phase 3: Focus groups

Seven SLTs participated in one focus group, and six dementia HCPs participated in the other (Table 4).

**Table 4:** Demographic of participant population for phase three, staff focus groups

	*n*	Age group (years)						Gender		Professional background			Experience with dementia				
		18–24	25–34	35–44	45–54	55–64	≥65	Female	Male	Nursing	Psychology	SLT	<1	1–5	6–10	11–15	>15
Speech & language therapists (SLT)	7		5	1		1		7				7	1	3	3		
Healthcare professionals	6	1	1		1	1	2	6		5	1			1	2	1	2

## Findings

Four main service delivery-related themes were identified: *dementia-related communication changes; accessing support for communication; identifying communication strategies;* and *service considerations*. Findings from all three study phases are presented together under each theme, to aid understanding of key issues from different perspectives.

### Theme 1: Dementia-related communication changes

All participant groups discussed the complexity of dementia-related communication changes. Their broad and varying nature has implications for service considerations and therefore will be briefly presented here.

Participants reported observing communication changes in PWD, especially Alzheimer’s Disease, which were typically related to word-finding and short-term memory loss:

*“Sometimes he wouldn’t know what you’re talking about, and naming of objects….” (Interview relative P209).*

As dementia progressed, participants reported broader and more severe cognitive communication changes, impacting information-processing, receptive language, conversation length and behaviour changes, with increased reliance on non-verbal communication. Participants commented on the impact of communication changes on the PWD, including frustration and potential relationship-breakdown early-on, then reduced engagement and participation as the disease progressed:

*‘I don’t say anything, and I struggle to listen, so it feels like work being with a group of people. Feels like an effort being with a group of people, whereas before it was my life’ (Interview PWD P206).*

PWD and their relatives highlighted fluctuations in communication difficulties:

*‘It varies tremendously, yes. I can’t find the right words some days. Other days, that same conversation would be totally different.’ (Interview PWD P201).*

All participant groups described communication changes as ‘significant’:

*‘I think communication difficulty is one of the most significant factors influencing quality-of-life for people living with dementia. It is often the reason there are “unmet needs”, which then get expressed in ways that can be distressing for all concerned, leading to breakdowns in relationships and care.’ (Survey participant).*

Participants associated communication changes with decreased engagement and increased social withdrawal. They highlighted the importance of supporting socialisation:

*‘Communication is not just your basic needs of food, drink, hunger, thirst, pain. It is those interaction elements of building…relationships for…emotional well-being’ (Focus group SLT P312).*

### Theme 2: Accessing support for communication

The survey results demonstrated prioritisation of dysphagia over communication input. The highest proportion of respondents (n = 32, 43%) reported that only roughly 1–20% of their previous year’s dementia caseload had received targeted communication input (the remainder having received swallowing input only) (Appendix 4).

In the local NHS Trust, where communication input was not commissioned, one HCP participant felt that communication support ‘takes a back seat’ (focus group HCP P308) and the need for it was underestimated. Other SLT focus group participants corroborated this:

*‘Mostly what we do as a service [is] just signpost to other services. We…aren’t commissioned to provide input for communication for PWD’ (Focus group SLT P313).*

They felt the lack of communication provision could subject patients to increased risk:

‘*It’s not just about dysphagia and immediate risk, but actually risks associated with…poor interaction and poor communication in terms of their emotional well-being.’ (Focus group SLT P312).*

One SLT felt that people did not receive support unless they ‘shout’ (focus group SLT P306) for it.

### Lottery

Phase one suggested that most services accept PWD for communication input (n = 48, 65%). 17 (23%) worked in services that did not accept communication referrals, 7 (9%) accepted referrals for certain dementia-subtypes only, and 2 respondents (3%) were unsure. For those that did not accept communication referrals, lack of commissioning was the main reason (n = 12, 71%).

Uneven care and the notion of a ‘postcode lottery’ were highlighted as fundamental issues by focus group participants, with staff describing going beyond their roles to help individuals:

*“What they’ve perhaps had in one area might be very different to another, I think it causes confusion to professionals but also to families as well.” (Focus group SLT P313).*

SLT focus group participants reported sometimes providing communication support despite not being commissioned, but the nature of this varied because there was not a standard pathway. This ranged from one-off communication assessments with advice and strategies, posting communication strategy information with assessment letters, signposting and providing communication advice on dysphagia-related visits:

*‘That’s only given if they are there doing a swallowing assessment, so if person doesn’t have dysphagia they won’t get that advice’ (Focus group SLT P311).”*

For one dyad, access to direct communication support was available, possibly due to living on a county border:

*‘We were referred to the group from the memory clinic [and] they put us forward for…speech therapy… Our consultant…said it wasn’t something that [the local Trust] pay for [and] because we were just over the border from [county] -obviously, [county] has got an allowance for it.’ (Interview relative P207).*

### Navigating services

Nearly all survey respondents (n = 71, 96%) felt that PWD had not been able to access the support they needed for communication. Barriers identified were: lack of referrals (n = 57, 77%) and awareness of provision (n = 33, 45%), and restrictive service criteria (n = 35, 47%).

Although not specifically focused as communication support, participants had found local or charity support groups helpful, especially for sharing experiences:

*“You can meet for a cup of tea and chat, people of a similar illness… See people of a similar problem to be able to chat and realise that you’re not the only person in the world, there are lots of other people have got it” (Interview relative P205).*

However, half of survey respondents (n = 37, 50%) were unaware of services that could provide communication support e.g. third-sector services. Phase two findings corroborated this, in that PWD and their relatives were also not necessarily aware of available support and services:

*‘No, none whatsoever. I didn’t even know they were there!’ (Interview relative P208).*

One relative also indicated that the loss of confidence experienced through communication changes had impacted the PWD’s participation in groups:

*‘We sat on a table just the two of us together. [She] didn’t feel comfortable about joining another table’ (Interview relative P203).*

Several participants discussed how specific communication services were challenging to find, but they had had positive experiences with other healthcare services or interventions which promoted social communication:

*“The [CST] sessions…they’re helping me short-term I would say…When I leave [CST] I feel better... That one-to-one conversation with her and what she does for me, it feels like she just makes me feel better” (Interview PWD P206).*

PWD and their relatives expressed a sense of abandonment, lack of communication between services, and feeling overwhelmed. This contributed to challenges finding and accessing appropriate support:

*‘I’m a tough, strong person, and I could really have done with some help earlier on... because I was lost and drowning. It was just from lack of information or lack of knowing where to go.’ (Interview relative P206).*

### Theme 3: Identifying communication strategies

HCPs and SLTs emphasised the importance of tailoring communication strategies to the individual, with implications for improving behavioural and psychological dementia symptoms:

*‘We get a lot of PWD who are agitated, and a lot of agitation is directly related to the poor communication on behalf of the staff with the residents.’ (Focus group HCP P310).*

HCPs noted that relatives of people with advanced dementia often relied on non-verbal communication and interpreting behavioural changes:

*‘Even though he was non-verbal really, with his speech…his non-verbal communication was still intact for his wife.’ (Focus group HCP P301).*

One dyad who had received communication input described therapy approaches such as co-developing a communication and memory prompt book with their SLT, which aimed to increase independence:

*“We’ve got a local map and a page with [pictures of] food in, because [he] finds that quite difficult to [communicate].” (Interview relative P207).*

Phase 2 participants who had not received formal communication support reported developing their own communication strategies. These included establishing motivating conversation topics, and adapting communication to support comprehension:

*‘So encourage the conversation, sort of thing [for example] yesterday we did seeds for the garden…and we discussed the seeds, and bits and pieces’ (Interview relative P201).*

*‘I’ve just learnt to communicate in a different way…there’s no rolling of eyes, you just have to say it like it is in a very black and white way. It’s just learning to navigate a new type of communication.’ (Interview relative P206).*

### Theme 4: Service considerations

Survey results suggested unstandardised communication support provision for this population. 54 (73%) respondents stated that they did not have a pathway for PWD requiring communication input. 19 of these respondents did not accept communication referrals for PWD; therefore, 35 (47%) respondents accepted these referrals, but did not have an established care pathway for them. Phase 3 participants reported challenges to formalising care pathways, including: variable presentations, stages and sub-types of dementia; compounding effects of additional diagnoses; cognitive impairments impacting patients’ ability to participate in therapy; need for individualisation of care; and lack of evidence.

One survey respondent suggested that national advice would help to improve care:

*‘It would be helpful for local services and commissioners if there were a national framework outlining benefits and expectations for communication provision for PWD, with particular reference to the role of SLTs.’ (Survey participant).*

### Estimating the need for communication input

Several focus group HCPs described not referring to SLT for communication support:

*‘I wouldn’t even think to refer for communication unless someone’s had a stroke or something like that.’ (Focus group HCP P303).*

However, focus group SLTs described receiving many ineligible referrals for communication support for PWD:

*“The number of referrals that we have to reject per week or month, we just have to say ‘sorry you don’t fit the box that we work in’” (Focus group SLT P306).*

SLT focus group participants emphasised the importance of further research to quantify unmet need and identify who would benefit most from communication input. They felt this would help inform commissioners and local services of service-level priorities, as well as the additional resources required to address these needs:

*“Without knowing the evidence-base, it’s like opening a can of worms, saying ‘ok we’ll accept every referral for communication in dementia’ – unknown numbers could be overwhelming” (Focus group SLT P306).*

### Embedding communication input locally

Focus group HCPs from local memory clinics reported that their commissioning as a diagnostic service limited the ongoing support they could provide. They explained that post-diagnostic care was passed to the voluntary sector, who advised on communication generally, but did not include SLT:

*“We don’t see people long enough to deliver therapeutic interventions…we are a diagnostic service…we’re giving medication but we’re not really helping in more practical ways...We hand on to the voluntary sector but they aren’t equipped to deal with this” (Focus group HCP P308).*

Participants in both focus groups discussed a need for joined-up working across local services, which could be difficult to manage. The need for a ‘continued’ service throughout the patient’s dementia diagnosis was highlighted:

*‘[The] ideal service would…Have joined-up care from the point of diagnosis…It would be a continued service…So that the person has support from day one to the very end’ (Focus group HCP P301).*

### Training and awareness needs

Communication interventions provided by survey participants (SLTs) tended to be communication skills training for families (n = 60, 81%). During interviews, relatives also expressed a desire for more information and support following diagnosis, including more tailored information on communication changes.

*“For the carer or the family, to really…understand what it is that we’re dealing with…[to] have a professional say, ‘Look, this is what’s going on in your mum’s head. This is why she’s having communication difficulties, and this is what you could do’.” (Interview relative P202).*

One relative also indicated that raising dementia awareness more widely may help to reduce stigma:

*‘I think the general ignorance amongst the general public…people have got to get a better understanding - not to take a wide berth of somebody that’s having communication problems.’ (Interview relative P208).*

Focus group participants also discussed the need for more staff awareness:

*‘I’m always amazed we go in and see someone is agitated and all the information is there…and they haven’t read it. Staggered by how little some people know.’ (Focus group HCP P310).*

Unmet training needs were reported as a barrier to providing communication support. SLTs and HCPs reported limited training in dementia-related communication difficulties:

*‘It should be integrated into…every level - health education framework for dementia services. I think communication could be built into the health education programme HEE’ (Focus group HCP P308).*

Despite the need, it was unclear who could provide awareness-raising and training, given the absence of a commissioned service. The SLT group suggested that utilising students and assistant psychologists could be a cost-effective way of trialling this initiative. However, this would require oversight and training from an experienced SLT.

### Service delivery format

SLT survey respondents who provided communication interventions for PWD reported primarily delivering therapy one-to-one (including dyadic therapy) (n = 55, 74%), rather than group therapy (n = 0), or a combination of one-to-one and group sessions (n = 9, 12%). Phase 2 participants agreed, as communication difficulties can impact group interactions:

*‘I think that if there’s a lot of people there, you start thinking, oh, can they understand what I’m saying? If you talk to somebody on their own, it’s easier for everybody.’ (Interview PWD P202).*

A common consideration was that, although remote support can improve access for some, it can also pose difficulties for others who are less familiar with technology or experience a decline in technical abilities:

*‘We are migrants to the digital scene…Not as savvy, digitally…But on the other hand, there are certain advantages in doing things digitally’ (Interview relative P203).*

## Discussion

Previous research has highlighted the complex, fluctuating and variable nature of dementia-related communication difficulties, as well as their quality-of-life impact [12, 19, 20, 22]. This study corroborates this from the viewpoints of PWD, their relatives, SLTs and dementia HCPs. This triangulation of perspectives adds weight and depth to the growing evidence-base. This study also considers the challenges of identifying and accessing appropriate support and highlights key services considerations. Participants from one NHS Trust were consulted, with a national survey of SLTs adding broader context to the findings.

Participants reported that a lack of communication provision/support impacted on quality-of-life of PWD and relatives, and increased patient risk. Dementia HCPs reported that communication breakdowns contributed to behavioural changes for PWD, necessitating more intensive care. This is in line with research, which suggests that unmet speech, language and communication needs in dementia can ultimately result in care crises, which can be challenging to navigate, have quality-of-life implications, and require additional resource and funding [21, 27, 33, 34]. These unmet needs can also increase risk of mental health consequences for PWD and caregivers. Unmet communication needs can hinder access to and communication with other health services, leading to additional unmet health needs, and perpetuating health inequalities for PWD [21].

Participants reported barriers to accessing communication support, primarily around lack of commissioning. Commissioning varies between localities, contributing to a national postcode lottery; most participants lived in an area where communication-focused SLT was not commissioned. However, even within these localities, provision varied, as people living on county borders were sometimes able to access communication support from neighbouring areas. The issue of a postcode lottery in post-diagnostic dementia support has been highlighted in previous research in Wales [35]. Additional barriers, such as a lack of referrals, specialist dementia SLTs, difficulties navigating services, and SLT role-awareness were also reported. Therefore, while commissioning was a key challenge, additional areas require consideration to improve access to communication support for PWD.

PWD and their relatives had identified and developed strategies to improve their ability to communicate through trial and error. This demonstrates the importance of an individualised approach to communication support provision, working closely with PWD and their relatives to co-develop resources and strategies, harnessing expertise by experience [36]. Survey respondents who had treated PWD generally reported the effectiveness of conversation-partner training with the PWD and a relative. This corroborates findings from Volkmer et al.’s [22] work which showed conversation-partner training was a promising intervention for primary progressive aphasia (a language-led dementia).

Combining national survey, patient and relative interview and staff focus group results permits reflection on potential avenues for service improvement locally and nationally. Locally, there are challenges estimating the level of need for communication input for PWD. Whilst HCPs working in memory clinic reported not generally referring PWD to SLT for communication support, SLTs reported receiving referrals. These may be coming from a variety of sources. A way of measuring the percentage of PWD attending memory services who might benefit from communication support, for example through an audit tool [37], would help determine whether provision of a service is indicated and feasible, and could provide evidence to support a business case.

This study’s findings indicate that there is not one clear pathway nationally for provision of communication support in dementia. Locally, post-diagnostic support for PWD is provided by third-sector organisations, without an SLT service. With the variable nature of dementia and variable UK service models, service providers need to think creatively about the best ways to incorporate communication input locally, taking a systems-approach [38]. For example, to explore with stakeholders whether SLT input would best sit in diagnostic memory clinic settings or as an in-reach service to third-sector organisations, to support PWD later in their diagnosis. Survey respondents suggested that national frameworks might be useful to support service provision. Frameworks such as the Allied Health Professionals Dementia Framework for Wales [39] may help guide the development of appropriate and accessible services.

Interview and focus group participants stated that continued, joined up care was important. Strengthening connections between memory clinic and post-diagnostic support services, for example SLT, may raise awareness of support avenues for PWD and improve referral rates to SLT services. Inter-service training sessions could help develop networks and relationships between staff which may positively impact joined up care provision. However, Reeves et al. [40] caution that despite some growth in the evidence base for the effectiveness of inter-professional education, further research is needed to clarify its benefits for patients. Another way to improve the continuity of care is through providing a named or central point of contact such as a key worker for the PWD and their relatives. An appointed contact person is a central recommendation to support access to services for people with dementia according to the best practice recommendations for people with dementia in the Europe Actifcare project [41]. Goeman, Renehan and Koch’s [42] systematic review found that components of the key worker role such as individualised education and support and collaborative, multi-disciplinary input positively impacted the quality-of-life of PWD.

Once a service to support communication is established, there are considerations for format and content. Study participants reported that face-to-face one-to-one communication support/therapy was desirable. However, its practical and financial feasibility in stretched healthcare services is questionable. Remote therapy may be more cost-effective, but evidence for its therapeutic effectiveness in dementia remains unclear [43]. Service models implemented would benefit from being co-developed with PWD/families/services to have greater chances of implementation success [44]. There may be risks of delivering a communication support service that is either one-size-fits-all or very limited, e.g. provision of a one-off advice sheet. A one-size-fits-all approach to dementia, a highly variable condition, may cause unnecessary anxiety or misunderstandings if not accompanied by consistent support [45]. Information regarding dementia should be communicated sensitively, recognising that some individuals may struggle to discuss its progression or be labelled with dementia-related terminology [46].

### Strengths and limitations

This study used triangulation of multiple data sources and perspectives, increasing the validity of the findings. It situated local service delivery within the national context, permitting reflection on implications for the local service and for other similar services. This study incorporated the perspectives of individuals living with dementia, a population often underrepresented in research. Additionally, it included the viewpoints of other stakeholders whose insights are crucial for implementing sustainable service improvements.

Although the study included the views of PWD, both as research participants and PPI members, only those who could provide informed consent and whose communication difficulties did not preclude them from participating in an interview, were eligible. Therefore, the experiences of people with more severe dementia and/or communication difficulties were not fully represented. The national survey sought to include a wide range of SLTs, and respondents were diverse in geographic location, experience and clinical setting. However, the voluntary nature of participation could introduce self-selection bias. SLTs with a specific interest in communication in dementia may have been more likely to complete the survey. Phase 3 focus groups explored staff views at one NHS Trust, providing interesting detail about service delivery in practice. Had resources allowed, it would have been useful to compare this NHS Trust with others to gather insights into different service delivery models.

## Conclusion

This study explored access to communication-focused SLT input for PWD. It considered multiple stakeholder perspectives locally and nationally. The findings demonstrate the impact of communication difficulties on quality-of-life for people affected by dementia and show the complexity of service provision in this area. Despite this complexity, joined-up and individualised post-diagnostic care when planning communication support for this population remains imperative. The presence of a considerable postcode lottery highlights the need for greater awareness and prioritisation of communication difficulties in dementia by commissioners. Given the prevalence of dementia, further research is required to determine the extent of unmet need. This can inform resource allocation, service eligibility and prioritisation criteria.

## Supplementary Material

aa-25-0134-File003_afaf150

aa-25-0134-File002_afaf150

## Research and Data Transparency and Availability:

Data are available from the authors upon reasonable request.
