# Supplementary material for: Access to communication support for community-dwelling people with dementia: A mixed methods study exploring local perspectives within the United Kingdom context
Source: Age Ageing. 2025 Jun 3;54(6):afaf150. doi: 10.1093/ageing/afaf150 (PMC12131240; doi:10.1093/ageing/afaf150)
Supplement: aa-25-0134-File003_afaf150 [file aa-25-0134-file003_afaf150.docx]

**Access to communication support for community-dwelling people with dementia: A mixed methods study exploring local perspectives within the United Kingdom context**

**Appendices**

**Contents**

**Appendix 1 – Survey Questions pdf – uploaded separately**

**p.2 Appendix 2 - Interview Topic Guide – PWD and relative**

**p.6 Appendix 3 – Focus Group Topic Guide**

**p.9 Appendix 4 – Survey Results**

**Appendix 2 – Interview Topic Guide – PWD and relative**

| Main questions | Sub questions |
| --- | --- |
| Introductory questions |  |
| We’re interviewing people about changes to their life following dementia diagnosis. In particular, we’re interested in speech and language changes in dementia. We’ll be asking you questions about communication as a whole. | |
| What does communication mean to you? | What do you understand by the word communication?  Prompts - Making yourself understood, expressing yourself, understanding others.  In your life, is communication important to you? In what ways? |
| Have you noticed any changes to your ability to communicate? | Some people notice they: get stuck on words, repeat phrases, have trouble finding the right words – have you noticed any of these?  People have also noticed other changes such as forgetting, saying unexpected things, losing track of a conversation, talking less, using gesture – have you noticed any of these? |
| How do these changes affect your day-to-day life? | Practical – e.g. making appointments, telephone, managing bills etc.  Social – spending time with friends and family, attending events/groups, phoning friends/family  Emotional – how you feel in yourself, confidence, independence |
| **Main question:** How have communication changes affected your quality of life? | How important is being able to communicate to you?  Being independent, doing things you used to do, enjoying life, relationships |
| We’re interested to know if anything has helped with these communication difficulties; have you received any help? | What has helped you with these difficulties?  How do you get around these difficulties?  Have you talked to anyone about these difficulties with communication? (any services?)  How easy was it to get help for the communication difficulties? At what stage were you offered/sought help?  Have you had any contact with speech and language therapy services? Yes – tell me more. |
| *How did the SLT (or other formal help) impact your quality of life?* | *By quality of life we mean: how you’ve been feeling, things you’ve been able to do, enjoying life*  *In what ways did it help?*  *How did it affect your practical, social, emotional areas?*  *How are things different now/what things are you doing differently now?* |
| Ending questions  What else might be helpful? | Content – training, one to one coaching,  When? Memory clinic? Later? One-off or ongoing?  How to access – GP, third-sector, memory clinic etc.  Format – face to face, online, telephone support, information packs, individual vs group. |
| Is there anything else you’d like to say in relation to communication? |  |

| **Main questions** | **Sub questions** |
| --- | --- |
| **Introduction to the interview:** | |
| We’re interviewing people about changes to their life following dementia diagnosis. In particular, we’re interested in *speech and language* changes in dementia. We’ll be asking you questions about your relative’s communication as a whole. | |
| **Introductory questions** |  |
| What does communication mean to you? | What do you understand by the word communication?  Prompts - Making yourself understood, expressing yourself, understanding others.  In your relative’s life, is communication important? In what ways?  For you and your relative together, is communication important? |
| Have you noticed any changes to your relative’s ability to communicate? | Some people notice they: get stuck on words, repeat phrases, have trouble finding the right words – have you noticed any of these?  People have also noticed other changes such as forgetting, saying unexpected things, losing track of a conversation, talking less, using gesture – have you noticed any of these? |
| How do these changes affect your relative’s day-to-day life?  How has it affected your life? | Practical – e.g. making appointments, telephone, managing bills etc.  Social – spending time with friends and family, attending events/groups, phoning friends/family  Emotional – how you feel in yourself, confidence, independence |
| **Main questions:** |  |
| How have communication changes affected your relative’s and your quality of life? | How important is communication to your relative?  How important is your relative’s communication to you?  Being independent, doing things you used to do, enjoying life, relationships |
| We’re interested to know if anything has helped with these communication difficulties; have you and relative received any help? | What has helped you/your relative with these difficulties?  How does your relative get around these difficulties?  Have you/your relative talked to anyone about these difficulties with communication? (any services?)  How easy was it to get help for the communication difficulties? At what stage were you offered/sought help?  Have you/your relative had any contact with speech and language therapy services? Yes – tell me more. |
| *How did the SLT (or other formal help) impact your relative’s and your quality of life?* | *In what ways did it help your relative?*  *In what ways has it helped you?*  *How has it affected the practical, social, emotional areas?*  *How are things different now/what things are you/your relative doing differently now?* |
| **Ending questions:** |  |
| What else might be helpful for you in terms of support with your relative’s speech, language and communication? | What would you like therapy to work on? – training for carers? one to one coaching? Working on speech directly?  When would you like to be offered support with speech and language? At memory clinic? Later? One-off or ongoing?  How would you like to access support for speech and language – via GP, third-sector, memory clinic?  What format would you like therapy to be in? – face to face, online, telephone support, information packs, individual vs group. |
| Is there anything else you’d like to say in relation to communication? |  |

**Appendix 3 – Focus Group Topic Guide**

| **Main questions** | **Sub questions/prompts** |  |
| --- | --- | --- |
| We’ve invited you all here today as you’ve had some contact with people with dementia as part of your work. We’re particularly interested in speech and language changes in dementia. We’re aware that some UK Trusts offer communication input to people with dementia and others are not commissioned to do this. We want to get your opinion on how we currently treat these needs in our service. | | |
| **Introductory questions** |  |  |
| What is your understanding of communication problems in dementia? | What have you noticed in your patients?  Speech, words, conversations, relationships, behavior  Have you received training around communication problems in dementia? | 5 mins |
| How much are communication difficulties a problem for the people with dementia you see? | Impact on:  Quality of life?  Everyday life?  Social connectedness and relationships?  Wellbeing?  How significant do you think this problem is for people with dementia? And their caregivers?  How many patients/caregivers? | ? refer to quotes from interviews with people affected by dementia |
| What does your service currently offer people with dementia who have communication difficulties? | Forward-referrals?  Signposting?  Direct advice?  Do you have a treatment plan for this patient group?  What does your service allow you to do? |  |
| **Main question:** To what extent do the services our NHS Trust offer meet the communication needs of people with dementia? | What is the main strength of what your service offers in relation to communication support?  Do you feel what you are able to offer is sufficient for people with dementia and caregiver?  How do you know if the service has met the needs of the patient, in terms of their communication? |  |
| What are the barriers and facilitators to providing communication support for this client group? | E.g. Lack of need  Time constraints  Resources  Lack of research  Lack of knowledge  Decision-makers  Funding | Add the factors brought up in interviews with people affected by dementia e.g. services liaising with each other |
| **Ending questions** |  |  |
| If you were re-designing your service, would you make any changes to the current pathways for supporting people with dementia and communication difficulties? | Why/why not?  What would these changes be?  What would you need in order to be able to make these changes? E.g. finance, support from above etc. |  |

**Appendix 4 – Survey Results**

| **Survey question** | | **Responses (n=74)** |
| --- | --- | --- |
| Please estimate how many patients with dementia you have had on your caseload over the year, where you have supported them with communication (not swallowing) | None | 7 |
|  | 1-19 | 37 |
|  | 20-49 | 15 |
|  | 50-99 | 4 |
|  | 100+ | 4 |
|  | Unclear | 7 |
| Does your service accept dementia patients for communication input? | Yes | 48 |
|  | No | 17 |
|  | Not sure | 2 |
|  | Certain dementias only | 7 |
| If no, why not? | Not commissioned | 12 |
|  | Insufficient funding (prioritising dysphagia) | 2 |
|  | Lack of specialist staff | 2 |
|  | Unsure | 1 |
| Are you aware of other services local dementia patients can access for communication support? | Yes | 37 |
|  | No | 27 |
|  | No response | 10 |
| Do you feel there are PWD who haven’t accessed the communication support services they need? | Yes | 71 |
|  | No | 0 |
|  | Unsure | 3 |
| If yes, what are some of the barriers? | Geographical location | 17 |
|  | Language barrier | 20 |
|  | Offered but declined | 17 |
|  | Service criteria | 35 |
|  | Lack of awareness of provision | 33 |
|  | Not referred to SLT | 57 |
|  | Stigma | 1 |
|  | Insufficient capacity of SLT team | 8 |
| Considering your caseload of dementia patients over the past year, roughly what proportion have had targeted communication intervention? | None | 9 |
|  | 1-20% | 32 |
|  | 21-40% | 10 |
|  | 41-60% | 10 |
|  | 61-80% | 2 |
|  | 81-100% | 10 |
|  | Unsure | 1 |
| Do you have an established care pathway for PWD who require communication input? | Yes | 18 |
|  | No | 54 |
|  | In development | 1 |
|  | Unsure | 1 |
