# Supplementary material for: Access to communication support for community-dwelling people with dementia: A mixed methods study exploring local perspectives within the United Kingdom context
Source: Age Ageing. 2025 Jun 3;54(6):afaf150. doi: 10.1093/ageing/afaf150 (PMC12131240; doi:10.1093/ageing/afaf150)
Supplement: aa-25-0134-File002_afaf150 [file aa-25-0134-file002_afaf150.pdf]

Section 1 - Your clinical background

2. My job title is: *

3. How many years post-qualification are you? Please select one option *

- ◯ Newly qualified
- ◯ 1-2
- ◯ 3-4
- ◯ 4-10
- ◯ 10+

4. What is your current NHS banding? Please select one option. *

- ◯ Newly qualified band 5
- ◯ Band 5
- ◯ Band 6
- ◯ Band 7
- ◯ Band 8
- ◯ Other

5. What healthcare service is your position funded by? Please note, if you're working privately and within the NHS, please only answer the subsequent questions in relation to your NHS role. *

- [ ] Acute health care
- [ ] Mental health care
- [ ] Primary care
- [ ] Charity/third sector
- [ ] Independent/private
- [ ] Other

6. Which of these categories most closely represents where you work geographically? *

- ( ) South West England
- ( ) South East England
- ( ) London
- ( ) East England
- ( ) West Midlands
- ( ) East Midlands
- ( ) Yorkshire and The Humber
- ( ) North West England
- ( ) North East England
- ( ) Ireland
- ( ) Wales
- ( ) Scotland
- ( ) Outside the UK

7. Have you ever seen a patient with confirmed or queried dementia within your NHS role? (if you answer 'no', the survey will end here) *

- ◯ Yes
- ◯ No

8. Have you seen patients with dementia for: *

- ◯ Swallowing
- ◯ Communication
- ◯ Both
- ◯ Other

9. Does your service accept dementia patients for communication input? *

- ◯ Yes
- ◯ No
- ◯ Not sure
- ◯ Other

10. If no, why not? *

11. Which setting have you seen patients with dementia in? Please tick all the boxes that apply. *

- ☐ Acute medical
- ☐ Inpatient rehabilitation unit
- ☐ Outpatients
- ☐ Community - domiciliary, ESD etc.
- ☐ Memory clinic
- ☐ Other: ____________

12. Are you working in a service that specialises in dementia and mental health, or a general adult service? *

- ○ Specialist dementia and mental health
- ○ General adult
- ○ Other: ____________

Section 2 - Patient demographics

In this and the remaining sections we are interested in dementia patients you have seen for communication input, not swallowing. We are interested in communication difficulties secondary to the dementia, not related to other causes e.g. stroke.

13. Dementia can include Alzheimer's disease, vascular dementia, Lewy-body dementia and primary progressive aphasia. Does your service accept referrals for all dementia types for communication input?

- ◯ Yes
- ◯ No
- ◯ Unsure
- ◯ [ ] Other

14. If no, please comment. E.g. if service provision varies for different subtypes which types do/don't have input?

[ ]

15. Please estimate how many patients with dementia you have had on your caseload over the year, where you have supported them with communication (not just swallowing). If you can remember the sorts of diagnoses that would be useful.

[ ]

16. Are you aware of other services local dementia patients can access for communication support? E.g. third-sector organisations. If yes, please describe.

[ ]

17. Do you feel there are people with dementia who haven't accessed the communication support services they need? *

- ○ Yes
- ○ No
- ○ Not sure

18. If yes, what are some of the barriers? (tick all that apply) *

- ☐ Geographical location
- ☐ Language barrier
- ☐ Offered but declined
- ☐ Service criteria
- ☐ Lack of awareness of provision
- ☐ Not referred to SLT
- ☐ Other: ______

19. Compared to previous years, do you feel the number of patients with dementia being seen for communication input on your caseload has:

- ○ Increased
- ○ Decreased
- ○ Stayed the same
- ○ N/a
- ○ Unsure

20. If you selected increased or decreased, please explain why you think this has happened

21. Which professionals mainly refer people with dementia to the speech and language therapy service where you work for communication input? (tick all that apply) *

- [ ] Psychiatrists
- [ ] Neurologists
- [ ] Geriatricians
- [ ] GPs
- [ ] Psychologists
- [ ] Other speech and language therapists
- [ ] Unsure
- [ ] N/a
- [ ] Other

22. Considering your caseload of dementia patients over the past year, roughly what proportion have had targeted communication intervention?

- ○ None
- ○ 1-20%
- ○ 21-40%
- ○ 41-60%
- ○ 61-80%
- ○ 81-100%
- ○ Other

23. For dementia patients referred for communication input, what were the symptoms that prompted the referral to your service? (you can select multiple options)

- ☐ Expressive aphasia
- ☐ Receptive aphasia
- ☐ Dysarthria
- ☐ Social communication difficulties
- ☐ None were referred for communication
- ☐ Other

24. When are the majority of these patients being referred to you for communication input? *

- ☐ Not referred to SLT
- ☐ Within a year of symptom onset (around diagnosis)
- ☐ 2-3 years post-diagnosis
- ☐ 4+ years post-diagnosis
- ☐ Unsure

25. Do you have an established care pathway for people with dementia who require communication input? *

- ○ Yes
- ○ No
- ○ Other

26. Please describe the care pathway:

Section 3: Time spent on management of this patient group

27. Does your service stipulate how long the communication intervention for dementia patients can last? For example, intermediate care often has a six-week intervention limit *

- ◯ Yes
- ◯ No
- ◯ N/A (service doesn't offer communication intervention)
- ◯ Other: ____________

28. If yes, please tick the approximate number of sessions offered

- ◯ None
- ◯ 1-2
- ◯ 2-3
- ◯ 4-6
- ◯ 6+
- ◯ Other: ____________

29. If no, how many sessions on average would you estimate you spend with an individual from this patient group working on communication?

- ◯ None
- ◯ 1-2
- ◯ 2-3
- ◯ 4-6
- ◯ 6+
- ◯ Other

30. If you were to re-design your service, ideally how much time would you like to spend with someone with dementia on functional interventions to support communication?

Section 4: Specific assessment and intervention approaches

31. Over the last year, which of the following assessment tools have you used for people with dementia?

- ☐ Comprehensive aphasia test (CAT)
- ☐ Psycholinguistic Assessment of Language Processing in Aphasia (PALPA)
- ☐ Boston Naming Test
- ☐ Cognitive Linguistic Quick Test (CLQT)
- ☐ Arizona Battery for Communication Disorders (ABCD)
- ☐ Barnes Language Assessment
- ☐ Mini Mental State Examination (MMSE)
- ☐ Informal impairment-based communication test/screen
- ☐ Informal interview with person/family on personal history/conversation
- ☐ Informal assessment or observation of communication in functional activities
- ☐ None or n/a
- ☐ Other

32. Over the last year, which of the following interventions have you used for people with dementia?

- ☐ Impairment-directed interventions including naming, semantic attributes etc.
- ☐ Impairment-directed interventions for speech production (e.g. dysarthria, AoS)
- ☐ Working on reading and writing
- ☐ Activity participation rehabilitation e.g. working on communication for cooking task
- ☐ Functional communication intervention e.g. practising total communication strategies
- ☐ Communication skills training for families/carers
- ☐ Communication skills training for staff (e.g. health professionals, carers)
- ☐ Communication aid provision and training e.g. communication book
- ☐ Introducing the person with dementia and family to social/support networks
- ☐ None or n/a
- ☐ Other

33. When delivering communication interventions to people with dementia and/or their relatives/carers, is this generally provided one-to-one or as group therapy?

- ○ One-to-one
- ○ Group
- ○ Both
- ○ None or n/a
- ○ Other

Final questions

34. Do you use any of the following as an outcome measure for patients with dementia when working on communication? (tick all that apply)

- ☐ Goal-setting/review
- ☐ TOMS
- ☐ Language assessments e.g. naming, picture description
- ☐ Self-rating scales
- ☐ Speech and language therapist rating scales
- ☐ Video recording of communication
- ☐ Quality of life measures e.g. Dem-QoL
- ☐ None or n/a
- ☐ Other: ____________

35. If applicable, please provide one example of a goal that you have set with a patient with dementia related to communication difficulties:

____

36. To what extent do you think communication difficulties impact on dementia patients' quality of life? Please give details and reasons for your response. *

____

37. Do you have any other comments relevant to this questionnaire? Please write them here.

This content is neither created nor endorsed by Microsoft. The data you submit will be sent to the form owner.

Microsoft Forms
